# Positive predictive value of a case definition for diabetes mellitus using automated administrative health data in children and youth exposed to antipsychotic drugs or control medications: a *Tennessee Medicaid* study

**DOI:** 10.1186/1471-2288-12-128

**Published:** 2012-08-24

**Authors:** William V Bobo, William O Cooper, C Michael Stein, Mark Olfson, Jackie Mounsey, James Daugherty, Wayne A Ray

**Affiliations:** 1 Department of Psychiatry, Vanderbilt University School of Medicine, 1500 21st Ave South, Suite 2200 Village at Vanderbilt, Nashville, TN, 37212, USA; 2 Department of Pediatrics, Vanderbilt University School of Medicine, Nashville, TN, 37212, USA; 3 Division of Clinical Pharmacology, Department of Internal Medicine, Vanderbilt University School of Medicine, Nashville, TN, 37212, USA; 4 Department of Psychiatry, Columbia University College of Physicians and Surgeons, New York, NY, USA; 5 Division of Pharmacoepidemiology, Department of Preventive Medicine, Vanderbilt University School of Medicine, Nashville, TN, 37212, USA; 6 Geriatric Research, Education and Clinical Center, Veterans Administration Tennessee Valley Health Care System, Nashville, TN, 37212, USA

**Keywords:** Type 2 diabetes, Computer case definition, Health administrative data, Validity, Positive predictive value

## Abstract

**Background:**

We developed and validated an automated database case definition for diabetes in children and youth to facilitate pharmacoepidemiologic investigations of medications and the risk of diabetes.

**Methods:**

The present study was part of an in-progress retrospective cohort study of antipsychotics and diabetes in Tennessee Medicaid enrollees aged 6–24 years. Diabetes was identified from diabetes-related medical care encounters: hospitalizations, outpatient visits, and filled prescriptions. The definition required either a primary inpatient diagnosis or at least two other encounters of different types, most commonly an outpatient diagnosis with a prescription. Type 1 diabetes was defined by insulin prescriptions with at most one oral hypoglycemic prescription; other cases were considered type 2 diabetes. The definition was validated for cohort members in the 15 county region geographically proximate to the investigators. Medical records were reviewed and adjudicated for cases that met the automated database definition as well as for a sample of persons with other diabetes-related medical care encounters.

**Results:**

The study included 64 cases that met the automated database definition. Records were adjudicated for 46 (71.9%), of which 41 (89.1%) met clinical criteria for newly diagnosed diabetes. The positive predictive value for type 1 diabetes was 80.0%. For type 2 and unspecified diabetes combined, the positive predictive value was 83.9%. The estimated sensitivity of the definition, based on adjudication for a sample of 30 cases not meeting the automated database definition, was 64.8%.

**Conclusion:**

These results suggest that the automated database case definition for diabetes may be useful for pharmacoepidemiologic studies of medications and diabetes.

## Background

The pronounced increase in adolescents and young adults of the incidence of type 2 diabetes [1,2] has stimulated interest in the epidemiology of diabetes in this population. Factors of interest include genetic characteristics [3,4], diet [5,6], lifestyle [7,8], environmental factors [9], and prescribed medications [10-12]. Given that type 2 diabetes is a chronic disease with serious health consequences [13-15], there is an urgent need to better understand its pathophysiology so that appropriate preventive measures can be devised in this vulnerable population. Although it is an important public health problem, medication-associated type 2 diabetes occurs infrequently. Clinical trials and prospective cohort studies are unlikely to have sufficient power or duration of follow-up needed to detect important inter-drug differences in type 2 diabetes risk, and will often exclude vulnerable populations such as children and youth.

Large automated databases of medical care encounters are therefore a valuable resource for observational studies of the epidemiology of medication-associated type 2 diabetes in children and youth. Database records of inpatient and outpatient medical care encounters allow efficient identification of newly diagnosed cases of diabetes for large populations. They are particularly valuable for studies of medications, as databases include computerized prescription records, which provide objective, detailed, reliable and relatively low-cost measures of drug exposure [16]. Database records of medical encounters may also allow identification of newly diagnosed cases of type 2 diabetes. However, these records are subject to misclassification [17,18], including identification of existing (rather than new-onset) diabetes, which may introduce bias that cannot be overcome using statistical adjustment or other data analytic techniques. A reliable computer case definition is therefore essential for conducting studies of newly diagnosed type 2 diabetes associated with medication exposure using automated databases.

Thus, we utilized a sample from an in-progress retrospective cohort study in Tennessee Medicaid enrollees 6–24 years of age who were treated with atypical antipsychotic drugs or control medications to develop and validate a case definition for newly diagnosed diabetes suitable for automated databases.

## Methods

### Sources of data

The automated database case definition was part of an in-progress retrospective cohort study of antipsychotics and the risk of type 2 diabetes among children and youth enrolled in Tennessee Medicaid [18]. Computerized Medicaid files include an enrollment file as well as files recording prescriptions filled at pharmacies, hospital admissions, outpatient visits, and long-term care residence. The Medicaid files have been augmented by linkage with computerized death certificates [18] and, since 1998, with the State Hospital Discharge File, a comprehensive, statewide database of hospital discharges and emergency department visits, which provides information occasionally missing from Medicaid files. These files permitted identification of both the study cohort and the medical care encounters used to identify potential diabetes cases [16,18].

Tennessee Medicaid is an expanded version of the joint federal-state Medicaid program that finances medical care for qualifying low income persons. Expansions in 1994 extended Medicaid to include previously uninsured persons. Tennessee Medicaid enrollees had no deductible, co-pay, or prescription limits for most of the study period. In 2005, a five prescription per month limit (of which only two prescriptions could be for brand-name drugs) was enacted. However, this requirement did not apply to children or youth under 21 years of age. Furthermore, diabetes medications (insulin preparations and oral hypoglycemics) and diabetic supplies were considered exempt (i.e., did not count toward the prescription limit) during the entire study period, and none of the diabetes drugs could be obtained over-the-counter. Thus, data were considered complete for diabetes prescriptions and supplies used as components of the diabetes computer case definition (discussed below).

Children and youth eligible for the cohort were 6 to 24 years of age and enrolled in Tennessee Medicaid at some time between 1 January 1996 and 31 December 2007. Cohort membership also required at least one year of prior enrollment (with respect to time zero, the first day in which the cohort member satisfied all of the inclusion and exclusion criteria for a recent initiator of study medications, as discussed below), during which there was medical care utilization and full prescription drug coverage (allowing lapses of ≤ 7 days), which maximized availability of data needed for study variables. The cohort excluded persons with life-threatening illness, institutional residence, diagnosed schizophrenia or related psychosis or other condition for which antipsychotics are the only recommended treatment, medical care indicating diabetes (including ICD-9-CM codes consistent with a diagnosis of diabetes and/or filled prescriptions for diabetes medications), pregnant women (because gestational diabetes might be misdiagnosed) or women with diagnosed polycystic ovarian syndrome (treated with oral hypoglycemics). Cohort members could not have been in the hospital in the past 30 days because Medicaid files do not include in-hospital medications.

The cohort consisted of eligible recent initiators of antipsychotics or other psychotropic drugs (mood stabilizers, attention-deficit hyperactivity disorder drugs, selective serotonin reuptake inhibitor and related antidepressants, benzodiazepines). Recent initiators filled a qualifying prescription for a study drug on a day of cohort eligibility, with no prescription fill for a study drug in the preceding 365 days. Cohort members could also have non-qualifying use of study drugs in the 90 days preceding the qualifying prescription to allow inclusion of patients starting a study drug shortly after a hospital discharge; however, for cohort members who filled a study drug prescription within this 90 day window, there had to be no filled prescriptions for study drug in the preceding 365 days. Follow-up began on the day following the prescription fill and ended with the end of the study, the 25th birthday, loss of enrollment, death, failure to meet study inclusion/exclusion criteria, or 365 days following the last day of current use of the study psychotropic drug.

The Vanderbilt Committee for the Protection of Human Subjects and the Tennessee Bureau of Medicaid and Department of Health approved the study.

## Automated Database Definition for Diabetes

**Figure 1:**
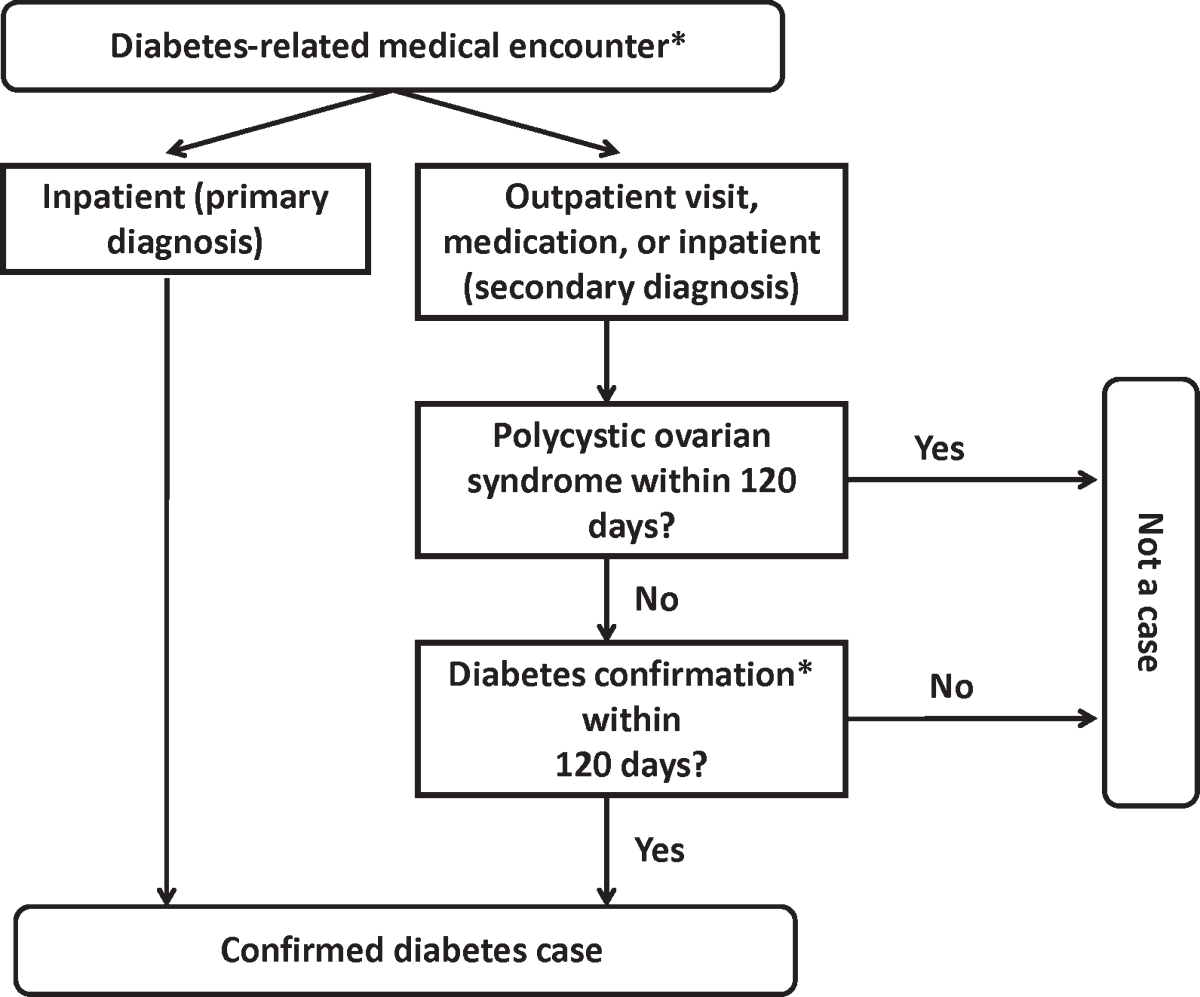
**Automated database case definition for diabetes mellitus.** * Confirmation of initial diabetes related medical encounters were required when such encounters were identified from secondary inpatient ICD-9-CM diagnosis codes, outpatient ICD-9-CM diagnosis codes, or filled prescriptions for diabetes medications (see Table 1 for complete definitions for diabetes-related medical encounters and diabetes confirmation). Confirmation was needed in order to limit potential misclassification. Medical encounters identified from ICD-9-CM diagnosis codes were confirmed by a subsequent prescription for diabetes medications. Medical encounters identified form prescriptions were confirmed by subsequent ICD-9-CM diabetes diagnosis codes or by a subsequent diabetes medication prescription with a procedure indicating diabetes management.

The primary automated database case definition (Figure 1) began with medical care encounters that indicated possible diagnosis or treatment of diabetes, termed *diabetes-related medical care encounters*. These encounters could be hospitalizations (tertiary or quaternary care settings), outpatient visits (primary or secondary care settings), or filled prescriptions for diabetes medications (Table 1). For each cohort member with such an encounter, we considered the first encounter during study follow-up.

**Table 1:** Definitions for automated database algorithm to identify diabetes

	***Inpatient-Primary***	***Inpatient-Secondary***	***Outpatient***^***†***^	***Prescription***^***‡***^
Diabetes-Related Medical Care Encounter*				
Definition	Primary discharge diagnosis of diabetes (ICD-9-CM codes of 250, 250.0, 250.1, 250.2, 250.3, 250.9)^§^	Inpatient stay with 1) a secondary or admission diagnosis for diabetes; or 2) a physician encounter with a primary diagnosis of diabetes during the *hospital stay period*, defined as the day prior to admission through the day following discharge.	Outpatient visit with a primary diagnosis of diabetes, excluding those during the hospital stay period.	Filled prescription for any diabetes medication, including insulin, insulin adjuncts (pramlintide), and oral hypoglycemics. There can be no diagnosis, primary or secondary, of polycystic ovarian syndrome in the interval [t_x_-120,t_x_ + 120]
Index date (t_x_) initial	Admission date or prior day if ED/ outpatient visit with diabetes diagnosis on that day	As for inpatient-primary	Day of visit	Day of prescription fill
*Additional Criteria for Diabetes Case*				
Exclusion^||^	None			
Confirmation^||^, primary definition	None	1. Diabetes medication prescription, or 2. Outpatient or inpatient (any) diagnosis	1. Diabetes medication prescription, or 2. Inpatient (any) diagnosis	1. Outpatient or inpatient (any) diagnosis, or 2. Subsequent prescription, and procedure indicating diabetes management^¶^, and no diagnosis absent/irregular menses.
Confirmation^||^, secondary definition	None	None	Glycosylated hemoglobin test (indicating possible diabetes management).	As above
Index date, final	If diabetes-related procedure^#^ in the interval [t_x_-29, t_x_-1] t_x_ is set to procedure date.			

*Does not include deaths as there were none with diabetes coded as an underlying cause of death for cohort members during the study period.

^†^Includes ED visits, but excludes case management services because these may not include patient assessment. Only primary outpatient diagnoses considered because secondary diagnoses occurred very infrequently in the absence of a primary diagnosis and a preliminary study showed they added little predictive value.

^‡^If both a prescription and other encounter on the same day, classified as a prescription encounter. Prescriptions with a concurrent diagnosis of polycystic ovarian syndrome were not considered as diabetes-related medical care encounters, given that this disease is frequently treated with oral hypoglycemics.

^§^Does not include 250.4-250.8, which are chronic complications of diabetes and thus unlikely to be present for newly diagnosed cases, particularly in a population of children/youth.

^||^Period for exclusion or confirmation is [t_x_-120, t_x_ + 120].

^¶^Diabetes management: HbA1c (glycated hemoglobin), glucose test strips, glucose monitor, insulin pump.

^#^Diabetes-related procedure: HbA1c, islet cell antibody test, insulin RIA, or metabolic panel.

If the encounter was a hospitalization with a primary discharge diagnosis of diabetes, the case definition was met. However, two additional steps were required for other types of encounters (Figure 1). First, cases with a diagnosis of polycystic ovarian syndrome within 120 days of the initial encounter were excluded. Polycystic ovarian syndrome was not uncommon in the study cohort, its symptoms overlap with those of diabetes, and it is often treated with oral antidiabetic drugs [19]. Second, confirmation of the initial encounter was required. Generally, a diagnosis would be confirmed by a subsequent prescription and a prescription by either a subsequent diagnosis of diabetes or by a subsequent diabetes medication prescription with a procedure indicating diabetes management (Table 1). This further confirmation was required because a single diagnosis frequently indicated a diagnostic workup and prescriptions in the absence of a diagnosis often were for polycystic ovarian syndrome.

Once the criteria for the automated database case definition were met, we assigned an index date. Generally, this was the date of the initial diabetes-related medical care encounter. Occasionally, the date was reset to that of an earlier visit (Table 1), corresponding to the clinical scenario in which the diagnosis was not made until after the results of a test were available.

The automated database definition also classified diabetes according to clinical subtype. The case was considered type 1 diabetes if there was at least one prescription for insulin within 120 days of the index date, with no more than a single prescription for an oral hypoglycemic in that interval. The single prescription for an oral agent was allowed because, on occasion, these drugs may be prescribed while awaiting the results of confirmatory testing for type 1 diabetes. Otherwise, the case was classified as type 2 diabetes.

The primary automated database case definition essentially identified diabetes treated with pharmacotherapy. It required both diagnosis of and treatment for diabetes, unless the patient was admitted to the hospital with a primary diagnosis of diabetes. In our sample, all hospitalized cases subsequently received medications. Consequently, the primary definition did not identify patients for whom diabetes was managed without medications. Thus, we also assessed a secondary automated database definition designed to better identify such cases. This definition (Table 1) accepted secondary inpatient diagnoses without further confirmation and relaxed the requirement for confirmation of an outpatient diagnosis with a prescription, requiring only a procedure for glycosylated hemoglobin testing (indicating diabetes management).

### Medical record review sample

Medical records for a sample of diabetes-related medical care encounters were reviewed to develop and validate the automated database case definition. The sample was drawn from members of the underlying cohort who had diabetes-related medical care encounters in the 15 county region within one travel day of Nashville. Only the first encounter for each cohort member was considered. The sample included all encounters that met the automated database case definition and a 60% sample of other encounters.

**Figure 2:**
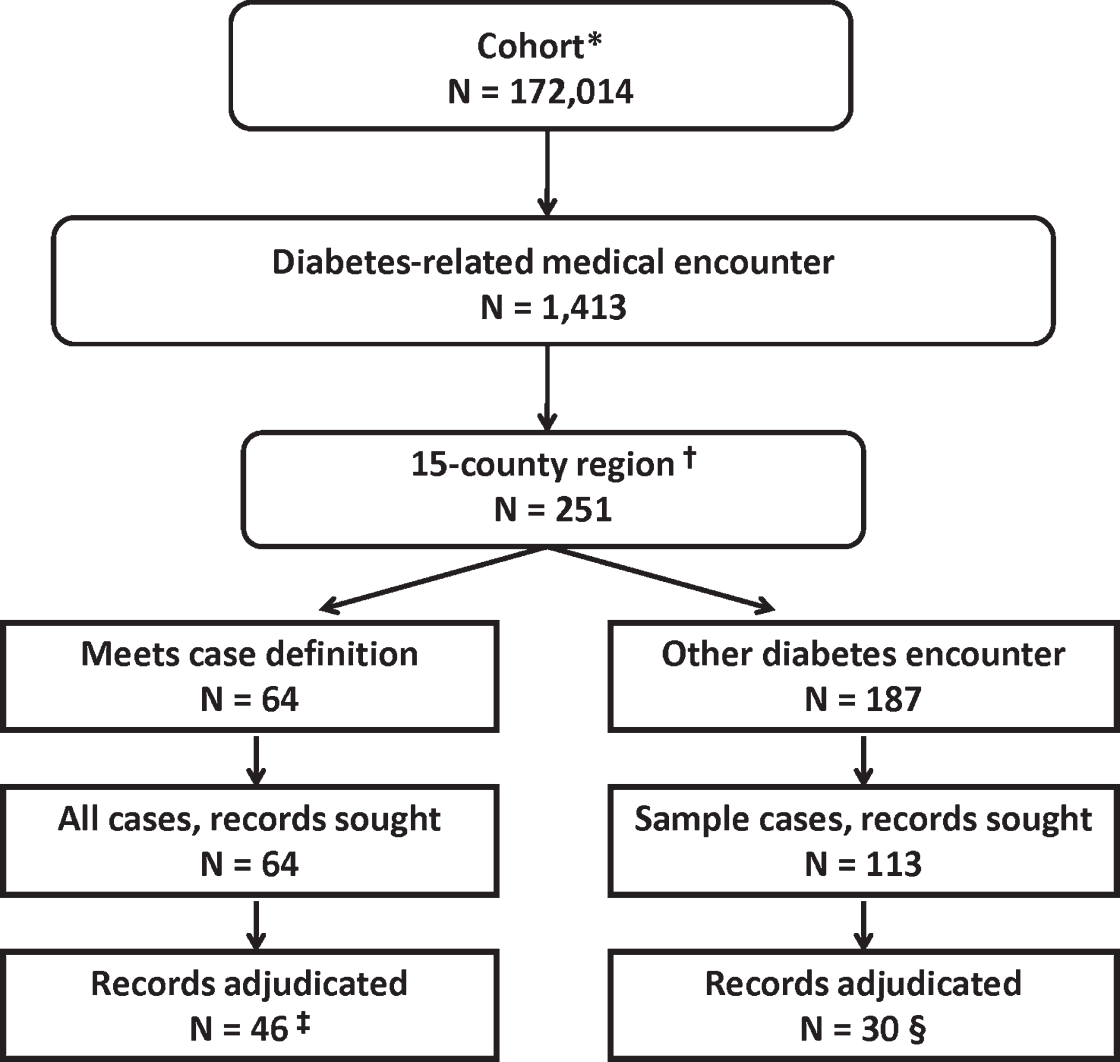
**Sample for validation of automated database case definition for diabetes mellitus.** *Preliminary version of the cohort. ^†^Counties (State of Tennessee) included: Cannon, Cheatham, Davidson, Dickson, Hickman, Lewis, Marshall, Maury, Montgomery, Robertson, Rutherford, Sumner, Trousdale, Williamson, Wilson. ^†^Counties (State of Tennessee) included: Cannon, Cheatham, Davidson, Dickson, Hickman, Lewis, Marshall, Maury, Montgomery, Robertson, Rutherford, Sumner, Trousdale, Williamson, Wilson. ^‡^There were 18 cases not adjudicated: 11--medical care provider identified, but patient record not located (most commonly for older records); 2--medical care provider not identified in Medicaid files; 2--medical care provider identified, but unable to visit (no longer practicing or relocated); 3--provider refusal. ^§^There were 83 cases not adjudicated: 45--medical care provider identified, but patient record not located (most commonly for older records); 20--medical care provider not identified in Medicaid files; 11--medical care provider identified, but unable to visit (no longer practicing or relocated); 4--provider refusal 3--patient records identified but lacked sufficient information for case adjudication.

The cohort utilized to develop the case definition included 172,014 members, of which 1,413 had a diabetes-related medical care encounter (Figure 2). Of these, 251 were in the 15 county region, of which 64 met the computer case definition and 187 did not. The medical record review sample included all of the former and 113 (60.4%) of the latter (Figure 2).

### Adjudication procedures

For sampled diabetes-related medical care encounters, study nurses reviewed records of all pertinent medical care within 365 days of the index date, focusing on those closest to the date of the diabetes-related encounter. They collected the results of laboratory tests, interventions undertaken, and, when appropriate, copies of medical records. All information was redacted to conceal patient identifying information. Each case was independently adjudicated by two investigators (WB,WC), masked to the exposure status of the patient, with disagreements resolved by a third reviewer (CMS).

The diagnostic criteria for diabetes required abnormal laboratory values for glycemic indices that exceeded standard cutoffs (Table 2) [20]. A non-fasting glucose value of ≥ 200 mg/dL was considered diabetes, absent an alternative recorded explanation for the elevated glucose. The adjudication did not use results of glycosylated hemoglobin tests, which recently have been accepted as a standard criterion for adults [21]. Possible diabetes was considered present if the medical record mentioned diabetes or hyperglycemia but there were no confirmatory laboratory tests (Table 2). Subthreshold hyperglycemia indicated an abnormally elevated glucose test that did not meet the criteria for diabetes (Table 2).

**Table 2:** Clinical criteria for diabetes, subthreshold hyperglycemia, and polycystic ovarian syndrome

**1. Diabetes mellitus (DM)**	**General criteria -- any of the following:**
	1. Fasting plasma glucose ≥ 126 mg/dL (7 mmol/L).
	2. Two-hour post-prandial glucose (following 75-g glucose load) ≥ 200 mg/dL (11.1 mmol/L).
	3. Signs or symptoms of diabetes mellitus (polyuria, polydipsia, nocturia, acanthosis nigricans, weight loss, obesity/weight gain) and random glucose ≥ 200 mg/dL (11.1 mmol/L).
***1.a. Type 1 DM***	***Subtype criteria – general DM criteria (above) met, and any of the following:***
	1. Type 1 DM diagnosis in medical record in conjunction with at least one of the following: (a) insulin treatment verified in medical record; (b) C-peptide concentration ≤ 0.2 nmol/L;* (c) positive islet cell (ICA) or glutamic acid decarboxylase antibody (GADA) assay; (d) no evidence of oral antidiabetic medication use. 2. Tentative type 1 DM diagnosis and any of the following: (a) insulin treatment verified in medical record; (b) no evidence of oral antidiabetic medication use.
***1.b. Type 2 DM***	***Subtype criteria –general DM criteria (above) met, and any of the following:***
	1. Type 2 DM diagnosis in medical record in conjunction with at least one of the following: (a) oral antidiabetic medication treatment verified in medical record; (b) diabetes-targeted lifestyle modification (dietary, physical activity, other weight loss) as primary diabetes treatment verified in medical record; (c) no evidence of insulin use.
***1.c. DM, unspecified***	***General criteria for DM met (see 1. above), but case does not meet sub-type criteria for either type 1- (see 1.a.) or type 2- (see 1.b.) DM.***
**2. Possible DM**	**Possible hyperglycemia or diabetes mellitus mentioned in record, but laboratory testing for diabetes mellitus not performed or results unknown/unavailable.**
**3. Subthreshold hyperglycemia**	**Laboratory testing for diabetes performed and results available, meeting any of the following:**
	1. Abnormally elevated fasting plasma glucose (100–125 mg/dL).
	2. Two-hour post-prandial glucose (following 75-g glucose load) 140–199 mg/dL.
	3. Abnormally elevated plasma glucose level, with or without clinical signs of diabetes mellitus, unable to verify fasting vs. non-fasting status.
**4. Polycystic ovarian syndrome**	**Any of the following:**
	1. Polycystic ovarian syndrome (PCOS) diagnosis documented in medical record, with or without clinical signs and symptoms consistent with PCOS (oligomenorrhea, amenorrhea, clinical or biochemical evidence of androgen excess, or polycystic ovaries diagnosed on ultrasonography or other imaging procedure).
	2. Tentative diagnosis of PCOS, with clinical signs and/or symptoms consistent with PCOS (as listed above).

*Based on diagnostic criteria used by Li et al. [36] and Bruno et al. [37].

Diabetes cases were further adjudicated as type 1/type 2/unspecified type. We also determined the time of first diagnosis. Patients with a diagnosis prior to the first day of cohort follow-up were considered prevalent cases.

### Statistical methods

The positive predictive value (PPV) of the diabetes case definition was calculated with 95% confidence intervals (CI) for binomial proportions using Wilson’s formula. Case confirmation from medical record review served as the gold standard. PPV calculations were conducted using STATA statistical software, version 11.0 (STATA Corporation; College Station, Texas, USA).

Sensitivity of the primary automated database case definition were estimated. This was expressed as a/(a + c), where (a) was the number of true cases identified by the definition and (c) was the number such cases that were missed [22]. The former was estimated as the number of cases in the catchment meeting the database definition times its positive predictive value. The number of missed cases was estimated as the number of diabetes-related medical encounters not meeting computer definition multiplied by the proportion of such cases that were true cases (see Additional file 1: Appendix 1 for details).

Specificity of the primary automated database case definition was also estimated. Specificity was expressed as d/(b + d) where (d) represents the estimated number of cases not meeting the computer case definition correctly identified as not being a diabetes case and (b) represents the estimated number of cases meeting the computer case definition misclassified as diabetes cases (see Additional file 1: Appendix 1 for details).

The study was funded by a grant from the Agency for Health Care Research and Quality, which had no role in study conduct or reporting. The listed authors were entirely responsible for study design, data analysis, manuscript preparation, and publication decisions; no other persons were involved. The first manuscript draft was written by the primary and senior authors, who vouch for the data and the analysis.

## Results

### Adjudication status

Of the 64 diabetes-related medical care encounters that met the primary automated database case definition, 46 (71.9%) were adjudicated (Figure 2). Fifteen of the 46 adjudicated cases satisfied the case definition of type 1 diabetes, while 31 satisfied the case definition for type 2 diabetes (Table 3). For the 76 total adjudicated cases (46 adjudicated cases that met the computer case definition; 30 adjudicated cases that did not meet the computer case definition [discussed below]), 77% were female and the mean age was 15 years.

**Table 3:** Adjudication status for diabetes-related medical care encounters meeting automated database definition for incident diabetes

	**Computer: Diabetes mellitus, any type**	**Computer: Type 1 diabetes mellitus**	**Computer: Type 2 diabetes mellitus**
	**Number (%)**	**Number (%)**	**Number (%)**
Adjudicated: Total	46 (100.0)	15 (100.0)	31 (100.0)
Adjudicated: Diabetes	41 (89.1)	14 (93.3)	27 (87.1)
Type 1	13 (28.3)	12 (80.0)	1 (3.2)
Type 2	25 (54.3)	2 (13.3)	23 (74.2)
Unspecified type	3 (6.5)	0 (0.0)	3 (9.7)
Adjudicated: Not Incident Diabetes	5 (10.9)	1 (6.7)	4 (12.9)
Prevalent diabetes	1 (2.2)	1 (6.7)	0 (0.0)
Subthreshold hyperglycemia	3 (6.5)	0 (0.0)	3 (9.7)
Polycystic ovarian syndrome	1 (2.2)	0 (0.0)	1 (3.2)

Of the 64 diabetes-related medication encounters that met the case definition, 18 records could not be adjudicated. The most common reason for non-adjudication was inability to locate a medical record for the patient, most commonly because these were older records that had not been retained (n = 11). Other reasons for non-adjudication included inability to identify a care provider in the Medicaid files (n = 2), inability to obtain records from providers that relocated or were no longer practicing (n = 2), and refusal of the provider to participate (n = 3). Of the 113 sampled diabetes-related medical care encounters that did not meet the case definition, 30 (26.5%) were adjudicated. For this group, 83 cases could not be adjudicated. The primary reason for non-adjudication also was inability to locate the patient’s medical record (n = 45); other reasons included inability to identify a care provider in the Medicaid files (n = 20), inability to obtain records from providers that relocated or were no longer practicing (n = 11), provider refusal (n = 4), and insufficient information for case adjudication (n = 3).

### Overall performance of case definition

Of the 46 adjudicated cases meeting the primary automated database definition for diabetes, 41 were adjudicated as diabetes with onset beginning during cohort follow-up (Table 3), resulting in a positive predictive value of 89.1% (95% CI 77.0, 95.3%). The five cases not adjudicated as diabetes included three cases of subthreshold hyperglycemia (Table 3; defined in Table 2), one case of diabetes with onset before t_0_, and one case of polycystic ovarian syndrome. The estimated sensitivity of the primary automated database definition for diabetes was 64.8% , while the specificity of the primary case definition was > 99%.

### Case definition performance by diabetes subtype

We also calculated the performance of the automated database case definition according to type of diabetes (Table 3). Of the 41 cases meeting the case definition adjudicated as diabetes, 13 were adjudicated as type 1, 25 were adjudicated as type 2 and for 3 the type was unspecified. The positive predictive value of the computer case definition for type 1 diabetes was 80.0% (95% CI 54.8, 93.0%) and that for type 2 was 74.2% (95% CI 56.8, 86.3%). When those cases for which type was unspecified were considered as type 2, the positive predictive value of the automated database definition for type 2 was 83.9% (95% CI 67.4, 92.9%).

### Cases that Did Not meet diabetes case definition

Of the 30 adjudicated cases that did not meet the primary automated database case definition, 5 (16.7%) were adjudicated as diabetes (Table 4). The other cases most commonly were possible diabetes (Table 2), subthreshold hyperglycemia, and polycystic ovarian syndrome (all treated with an oral hypoglycemic). The five confirmed cases had index diabetes-related medical care encounters that were outpatient visits (four cases) or secondary inpatient (one case) diagnoses; none was identified from a filled prescription.

**Table 4:** Adjudication status for diabetes-related medical care encounters not meeting the automated database definition for incident diabetes, by type of medical encounter

	**Inpatient, secondary diagnosis**	**Outpatient diagnosis**	**Filled prescription**	**Any medical encounter type**
	**Number (%)**	**Number (%)**	**Number (%)**	**Number (%)**
Adjudicated: Total	4 (100.0)	11 (100.0)	15 (100.0)	30 (100.0)
Adjudicated: Diabetes	1 (25.0)	4 (36.4)	0 (0.0)	5 (16.7)
Adjudicated: Not Diabetes	3 (75.0)	7 (63.6)	15 (100.0)	25 (83.3)
Prevalent diabetes	0 (0.0)	0 (0.0)	1 (6.7)	1 (3.3)
Possible diabetes	1 (25.0)	1 (9.1)	5 (33.3)	7 (23.3)
Subthreshold hyperglycemia	1 (25.0)	4 (36.4)	2 (13.3)	7 (23.3)
Polycystic ovarian syndrome	0 (0.0)	0 (0.0)	6 (20.0)	6 (20.0)
Laboratory test, rule-out	0 (0.0)	1 (9.1)	1 (6.7)	2 (6.7)
Miscoded diagnosis	1 (25.0)	1 (25.0)	0 (0.0)	2 (6.7)

### Performance of secondary diabetes case definition

We also assessed the performance of a secondary automated database case definition designed to better identify diabetes not treated with medications (Additional file 1: Appendix Table 1 and Additional file 1: Appendix Table 2; Additional file 1: Appendix Figure 1). The positive predictive value of this definition was 75.9% (95% CI 63.5, 85.0%) and the estimated sensitivity was 81.1% (95% CI 54.4, 73.9%).

## Discussion

We developed an algorithm to identify newly diagnosed cases of diabetes in children and youth that utilized automated database medical care encounter records. The primary automated database case definition, which generally required both diagnosis of and pharmacotherapy for diabetes, was validated by review of medical records with application of an objective standard for diabetes. In the sample studied, the positive predictive value for definite/probable diabetes was 89% and the case definition reliably distinguished between type 1 and type 2 diabetes. The estimated sensitivity was 65%. A secondary definition that better captured diabetes not treated with medications had a positive predictive value of 76% and an estimated sensitivity of 81%.

The availability of a valid computer case definition of new-onset type 2 diabetes is crucial for conducting pharmacoepidemiologic studies of type 2 diabetes as a study endpoint using automated databases. Automated databases may be the only efficient means of quantifying type 2 diabetes risk associated with specific drug exposures, given how infrequently it occurs. However, there are several challenges to conducting pharmacoepidemiologic studies using automated databases. Among the most serious of these is the potential for bias from endpoint misclassification due to coding errors or other problems [16,17]. Most automated databases, including the one used in our study, include medical encounter and healthcare service utilization data that were not collected specifically for research purposes. As such, the quality of the collected data may vary considerably [17]. In one study that used ICD-9 diagnosis codes from one or more outpatient records in the U.S. Indian Health Service Facility Database to estimate the prevalence and incidence of diabetes in Navajo youth, a diagnosis of diabetes was confirmed in less than 50% of cases [23]. The primary reason for misclassification was coding errors.

In our study, the most common source of misclassification was subthreshold hyperglycemia, accounting for 6% and 14% of adjudicated cases that respectively met the primary or secondary case definitions. These cases had an abnormal glucose laboratory value that was below the standard cutpoint for diabetes. This may represent in part the treatment of “prediabetes” [24], an increasingly common, yet controversial, trend among adults [25]. Although such cases might reflect adverse metabolic effects of medications, such as increased weight, we nevertheless considered them as false positives, given that the pathophysiology of drug-induced diabetes is incompletely understood [26].

We chose positive predictive value as the primary measure of algorithm performance based on our objective to develop and validate a computer case definition to facilitate pharmacoepidemiologic investigations of medications and the risk of new-onset diabetes. Determining sensitivity would quantify performance of our case definition only for those already known to have new-onset diabetes. In automated database studies, suspected (not established) cases of new-onset diabetes would be first identified. The positive predictive value is an ideal measure under this circumstance, as it represents the proportion of true cases among those identified by the computer algorithm as potential cases. Our results suggest that a high proportion of potential cases identified by our algorithm will be true cases.

Prior studies of the association between medications and new-onset diabetes in adults [27,28] have utilized validated case definitions based upon both diabetes diagnoses and prescriptions, which have been found to have good positive predictive value [29]. However, there are important differences for children and youth. First, type 1 diabetes has a much higher incidence. Distinguishing type 2 diabetes, the primary concern with respect to medication effects, must be considered for studies of younger populations. Distinguishing between type 1 and type 2 diabetes in the young is complicated, however, by the ever-increasing epidemic of overweight/obesity and other risk factors for insulin resistance in this population [30], which may result in misdiagnosis or substantial delays in arriving at a definitive diagnosis [31]. These factors make it difficult to differentiate type 1 and type 2 diabetes in youth using administrative data. Administrative data algorithms that rely on ICD-9 or −10 diagnosis codes for detecting diabetes in children or youth have been shown to capture type 1 diabetes more reliably than type 2 diabetes cases [32,33]. Although there are separate ICD-9-CM diagnostic codes for each type of diabetes, we and others [33] have found these to have little predictive value. The most reliable way to identify type 2 diabetes in our sample was to remove cases with a filled prescription for insulin, in the absence of repeated oral hypoglycemic prescriptions. Another unique aspect of populations with large numbers of women of reproductive age is the occurrence of polycystic ovarian syndrome, which, in our sample, often was indicated by a prescription for an oral hypoglycemic without a diagnosis of diabetes. In a recently published retrospective cohort study of diabetes incidence in children and adolescents that used antidiabetic drug prescriptions as a proxy for the disease itself, 22% of children who were prescribed metformin received the drug for treatment of polycystic ovarian syndrome [34].

Our primary automated database case definition focused on diabetes treated with pharmacotherapy, an important clinical consideration given that many individuals with type 2 diabetes will be treated with lifestyle interventions alone [35]. Our focus on medically-treated diabetes may have resulted in approximately one-third of the estimated total cases in the study sample being missed. Thus, we also assessed a secondary definition designed to better detect diabetes that was not treated with medications. The estimated sensitivity of this definition improved to 81%; however, the positive predictive value fell from 89% to 76%. The drop in the positive predictive value was primarily due to the misclassification of subthreshold hyperglycemia (14% of adjudicated cases) as diabetes.

### Limitations

The automated database case definition was developed and validated in a single sample that consisted of Tennessee Medicaid enrollees who had recently initiated therapy with a psychotropic medication. Although the algorithm has face validity, over-fitting is possible. Given these limitations, the performance of our algorithm in this cohort may not be generalizable to other patient populations, including Medicaid populations in other states and in non-psychiatric populations. Further investigations in other clinical settings and populations are needed.

We were unable to abstract and adjudicate 100% of all records sought that met the computer case definition of diabetes. Bias in our PPV estimate would be possible, particularly if there were systematic differences in the accuracy or quality of ICD-9 diagnosis coding, patterns of clinical practice, or clinical documentation between records that could be abstracted and those that could not. Under these circumstances, one would be less reassured that abstracted cases were representative of the source population. We were able to abstract and adjudicate nearly 72% of records for cases that met the diabetes computer case definition, and the most common reason for not being able to abstract such records was that records were too old to be retained by the practices. While these points provide some reassurance, we cannot exclude completely the possibility that systematic differences between abstracted and non-abstracted cases could have occurred, thus introducing potential bias in our PPV estimate.

Our estimate of the sensitivity of the automated database case definition relied upon adjudication of a sample of cases with diabetes-related medical care encounters that did not meet the database definition. We were only able to adjudicate 27% of the potential cases in this sample. The primary reason for the low rate of adjudication was that most of these consisted of a single prescription or outpatient diagnosis. In this circumstance, we often were unable to find the record for the individual patient, most commonly because these were older records that had not been retained in the practice. However, given that diabetes is a chronic disease, it seems unlikely that these isolated encounters represent true cases.

Finally, the “gold standard” for diabetes in the study sample was based upon review of medical care provider records. Although laboratory values were required for adjudication of a case as diabetes [36,37], standardized fasting glucose or glucose challenge tests were not uniformly obtained.

## Conclusion

We developed and validated an automated database case definition for newly diagnosed diabetes. The definition had an overall positive predictive value of 89% for definite/probable diabetes and could distinguish between type 1 and type 2 diabetes. If our findings are replicated in other settings, our algorithm could be a useful endpoint for pharmacoepidemiological studies that focus on the risk of medication-associated new-onset diabetes mellitus.

## Competing interests

This study was supported in part by the Agency for Healthcare Research and Quality (AHRQ), Centers for Education and Research on Therapeutics (HS1-0384), and CERT consortium grant (5U18HS017918-02). WVB is supported by a National Institute of Mental Health grant K23MH087747. The funders had no role in study design, data collection and analysis, decision to publish, or preparation of the manuscript. The study authors have not had in the previous 12 months a relevant duality of interest to disclose.

## Authors’ contributions

WVB, WOC and WAR conceived and designed the study. WVB, WOC, JM and JD researched data. WOC and JM participated in the coordination of the study. WVB and WAR drafted the manuscript. All authors contributed to, read, and approved the manuscript in its final version.

## Pre-publication history

The pre-publication history for this paper can be accessed here:

http://www.biomedcentral.com/1471-2288/12/128/prepub

## Supplementary Material

Additional file 1 **Appendix 1.** Calculation of Sensitivity and Specificity Estimates; Appendix Table 1. Secondary Automated Database Diabetes Definition: Adjudication status for diabetes-related medical care encounters meeting automated database definition for incident diabetes. Appendix Table 2. Secondary Automated Database Diabetes Definition: Adjudication status for diabetes-related medical care encounters not meeting the automated database definition for incident diabetes, by type of medical encounter. Appendix Figure 1. Secondary Automated Database Diabetes Definition: Validation sample. Click here for file
