# Supplementary material for: Positive predictive value of a case definition for diabetes mellitus using automated administrative health data in children and youth exposed to antipsychotic drugs or control medications: a Tennessee Medicaid study
Source: BMC Med Res Methodol. 2012 Aug 24;12:128. doi: 10.1186/1471-2288-12-128 (PMC3500229; doi:10.1186/1471-2288-12-128)
Supplement: Additional file 1 — Appendix 1. Calculation of Sensitivity and Specificity Estimates; Appendix Table 1. Secondary Automated Database Diabetes Definition: Adjudication status for diabetes-related medical care encounters meeting automated database definition for incident diabetes. Appendix Table 2. Secondary Automated Database Diabetes Definition: Adjudication status for diabetes-related medical care encounters not meeting the automated database definition for incident diabetes, by type of medical encounter. Appendix Figure 1. Secondary Automated Database Diabetes Definition: Validation sample. [file 1471-2288-12-128-S1.doc]

**Supporting Information**

The Appendix provides additional detail for the study methods and findings.

**Appendix 1. Calculation of Sensitivity and Specificity Estimates**

**Sensitivity.** We estimated the sensitivity of the primary automated database case definition. This can be expressed as a/(a+c), where (a) is the number of true cases identified by the case definition and (c) is the number of such cases that were missed. The former (a), was estimated as 64 x 0.89 (the number of cases in the catchment meeting the database definition multiplied by its PPV), or 57. The number of missed cases (c) was estimated as 187 (diabetes-related medical encounters not meeting the computer case definition) x 5/30 (the proportion of such cases that were true cases), or 31. Thus, the estimated sensitivity was 57/(57+31) = 0.648, or 64.8%.

**Specificity.** We also estimated the specificity of the primary automated database case definition, expressed as d/(b+d), where (d) represents the estimated number of cases not meeting the computer case definition correctly identified as not being a diabetes case, and (b) represents the estimated number of cases that meet the computer case definition but are incorrectly identified as diabetes cases (Figure). From the total cohort of 172, 014 persons (a+b+c+d), a total of 334 cases met the diabetes computer case definition. Therefore, (a+b) = 334. The estimated number of true positive cases (a) can be estimated by multiplying PPV x (a+b), or 0.89(334) = 298. The estimated number of false-positives (b) was obtained by subtracting (a) from (a+b), or 334 – 298 = 36. The estimated sensitivity, a/(a+c), was 0.648, as noted above. Therefore, (a+c) = a/0.648, or 298/0.648 = 460 (rounded to nearest whole number). The estimated number of false-negatives (c) was obtained by subtracting (a) from (a+c), or 460 – 298 = 162. To obtain the estimated number of true negatives (d), we subtracted (a), (b), and (c) from (a+b+c+d), or 172,014 – 298 – 36 – 162 = 171,518. Thus, the estimated specificity was 171,518/(36 + 171,518) = 0.9998, or 99.98%.

**Appendix Table 1. Secondary Automated Database Diabetes Definition: Adjudication status for diabetes-related medical care encounters meeting automated database definition for incident diabetes.**

**Appendix Table 2. Secondary Automated Database Diabetes Definition: Adjudication status for diabetes-related medical care encounters not meeting the automated database definition for incident diabetes, by type of medical encounter.**

**Appendix Figure 1. Secondary Automated Database Diabetes Definition: Validation sample.**

*Preliminary version of the cohort.

^†^Counties (in Tennessee) included: Cannon, Cheatham, Davidson, Dickson, Hickman, Lewis, Marshall, Maury, Montgomery, Robertson, Rutherford, Sumner, Trousdale, Williamson, Wilson.

^‡^The 12 cases for which records were not sought were those that did not meet the primary automated database definition and which were not in the sample of other cases.

^§^ There were 26 cases not adjudicated: 15--medical care provider identified, but patient record not located (most commonly for older records); 5--medical care provider not identified in Medicaid files; 3--medical care provider identified, but unable to visit (no longer practicing or relocated); 3--provider refusal.

^||^ There were 75 cases not adjudicated: 41--medical care provider identified, but patient record not located (most commonly for older records); 17--medical care provider not identified in Medicaid files; 10--medical care provider identified, but unable to visit (no longer practicing or relocated); 4--provider refusal 3--patient records identified but lacked sufficient information for case adjudication.

Appendix Table 1. Secondary Automated Database Diabetes Definition: Adjudication status for diabetes-related medical care encounters meeting automated database definition for incident diabetes.

|  | Computer: Diabetes mellitus, any type | Computer: Type 1 diabetes mellitus | Computer: Type 2 diabetes mellitus |
| --- | --- | --- | --- |
|  | Number (%) | Number (%) | Number (%) |
| Adjudicated: Total | 58 (100.0) | 15 (100.0) | 43 (100.0) |
|  |  |  |  |
| Adjudicated: Diabetes | 44 (75.9) | 14 (93.3) | 30 (69.8) |
| Type 1 | 14 (24.1) | 12 (80.0) | 2 (4.7) |
| Type 2 | 27 (46.6) | 2 (13.3) | 25 (58.1) |
| Unspecified type | 3 (5.2) | 0 (0.0) | 3 (7.0) |
| Adjudicated: Not Diabetes | 14 (24.1) | 1 (6.7) | 13 (30.2) |
| Prevalent diabetes | 1 (1.7) | 1 (6.7) | 0 (0.0) |
| Possible diabetes | 1 (1.7) | 0 (0.0) | 1 (2.3) |
| Subthreshold hyperglycemia | 8 (13.8) | 0 (0.0) | 8 (18.6) |
| Polycystic ovarian syndrome | 1 (1.7) | 0 (0.0) | 1 (2.3) |
| Laboratory test, rule-out | 1 (1.7) | 0 (0.0) | 1 (2.3) |
| Miscoded diagnosis | 2 (3.4) | 0 (0.0) | 2 (4.7) |

**Appendix Table 2. Secondary Automated Database Diabetes Definition: Adjudication status for diabetes-related medical care encounters not meeting the automated database definition for incident diabetes, by type of medical encounter.**

|  | Outpatient diagnosis | Filled  prescription | Any medical encounter type |
| --- | --- | --- | --- |
|  | Number (%) | Number (%) | Number (%) |
| Adjudicated: Total | 3 (100.0) | 15 (100.0) | 18 (100.0) |
|  |  |  |  |
| Adjudicated: Diabetes | 2 (66.7) | 0 (0.0) | 2 (11.1) |
|  |  |  |  |
| Adjudicated: Not Diabetes | 1 (33.3) | 15 (100.0) | 16 (88.9) |
| Prevalent diabetes | 0 (0.0) | 1 (6.7) | 1 (5.6) |
| Possible diabetes | 1 (33.3) | 5 (33.3) | 6 (33.3) |
| Subthreshold hyperglycemia | 0 (0.0) | 2 (13.3) | 2 (11.1) |
| Polycystic ovarian syndrome | 0 (0.0) | 6 (40.0) | 6 (33.3) |
| Laboratory test, rule-out | 0 (0.0) | 1 (6.7) | 1 (5.6) |

**Appendix Figure 1. Secondary Automated Database Diabetes Definition: Sample for validation of automated database case definition for diabetes.**
